# Listeners are better at predicting speakers similar to themselves

**DOI:** 10.1016/j.actpsy.2020.103094

**Published:** 2020-07

**Authors:** Lauren V. Hadley, Nina K. Fisher, Martin J. Pickering

**Affiliations:** a Hearing Sciences - Scottish Section, University of Nottingham, United Kingdom of Great Britain and Northern Ireland; b Department of Psychology, University of Edinburgh, United Kingdom of Great Britain and Northern Ireland

**Keywords:** Prediction, Simulation, Speech style, Turn-taking, Conversation

## Abstract

Although it takes several hundred milliseconds to prepare a spoken contribution, gaps between turns in conversation tend to be much shorter. To produce these short gaps, it appears that interlocutors predict the end of their partner's turn. The theory of prediction-by-simulation proposes that individuals use their own motor system to model a partner's upcoming actions by referring to prior production experience. In this study we investigate the role of motor experience for both predicting a turn-end and producing a spoken response by manipulating the similarity of heard speech to participants' own production style. We hypothesised that they would be better at predicting, and initiating responses to, speech produced in the style they speak themselves. Participants recorded a series of questions in two sessions, and several months later they listened to their own speech and that of a stylistically similar and a stylistically dissimilar participant (as assessed by independent raters). Participants predicted the end of 60 of these questions by pressing a button, and for the remaining 60 questions, by producing a spoken response. An analysis of response times showed that participants' button-press responses were faster for utterances spoken by themselves and by a stylistically similar partner, than for utterances spoken by a stylistically dissimilar partner. We conclude that simulation facilitates prediction of similar speakers.

## Highlights

• Prediction-by-simulation proposes that prediction is based on one's motor experience.
• If this is the case, predictions should be better for people more like oneself.
• We tested if speech-style similarity affected prediction during speech listening.
• Similarity led to better predictions of turn-ends, but not quicker turn-taking.
• We argue simulation facilitates prediction of similar speakers.

## 1 Introduction

Most research on speaking and listening has focused on language used by an isolated individual, despite the primary site of language use being conversation (Clark, 1996; Pickering & Garrod, 2004). Conversing with others is a highly complex task, and requires interlocutors to rapidly alternate between speaking and listening. Such rapid alternation raises an issue for interlocutors that does not occur in monologue – how do they determine when their partner is about to finish speaking, so that they are able to take over? In this paper, we investigate the possibility that they do so by simulating the speaker using the mechanisms that they use to speak themselves. If this is the case, they should be more successful the more similar their speech style is to the person they are interacting with. We now consider turn-end prediction in more detail, before reporting our experiment.

### 1.1 Predicting when the speaker will finish

Interlocutors are quite successful at such turn-taking, with the modal interval between turns being as short as 200 ms (Stivers et al., 2009). But it takes at least 600 ms to prepare to utter a single word (Indefrey & Levelt, 2004) and more than a second to prepare longer utterances (Griffin & Bock, 2000). Therefore, it appears likely that interlocutors regularly predict their partner's turn-end so that they can begin to prepare their response in time (Levinson, 2016). But how do they make such predictions?

Comprehenders make two types of prediction: what speakers will say, and when they will say it (Corps, Gambi, & Pickering, 2018). A great deal of research has focused on the former, and has found that they predict meaning (e.g., Altmann & Kamide, 1999), grammar (e.g., Van Berkum, Brown, Zwitserlood, Kooijman, & Hagoort, 2005), and sound (e.g., DeLong, Urbach, & Kutas, 2005). Such predictions facilitate comprehension, because they allow comprehenders to conduct some relevant processing ahead of time, and in particular help them to prepare appropriate responses.

A smaller body of research has asked how comprehenders predict speakers' timing, and in particular how comprehenders predict when they will finish speaking. There is some evidence that comprehenders use their understanding of what a speaker is saying to predict when the speaker's turn will finish. De Ruiter, Mitterer, and Enfield (2006) had participants listen to utterances and press a button when they expected them to end. Responses occurred earlier, and further from the actual end of the utterance, for utterances containing unintelligible words than intelligible words (when contour and envelope information were matched). They did not find that comprehenders were affected by the presence of pitch information, but other findings suggest that they do use prosody (Bögels & Torreira, 2015).

Other studies have directly considered whether predictable content improves turn-end prediction, but with rather mixed results. In one study, participants responded earlier for utterances containing predictable than unpredictable final words (Magyari, Bastiaansen, de Ruiter, & Levinson, 2014). This finding was replicated in another study showing earlier responses to questions when the final word was rendered predictable by a constraining context compared to an unconstraining one (Corps, Pickering, & Gambi, 2019). However, there was no effect of predictability on response precision (i.e., absolute distance from stimulus offset). Finally, a third study found that participants verbally answered yes/no questions earlier when they had predictable rather than unpredictable endings, but did not respond earlier when responding with a button-press, and did not show improved response precision (Corps, Crossley, Gambi, & Pickering, 2018).

### 1.2 Prediction-by-simulation

Much evidence suggests that people can predict other people's behaviour using their own motor system (Wolpert & Kawato, 1998). In other words, they engage some of the mechanisms that they use to perform such behaviours themselves (e.g., Wilson & Knoblich, 2005), covertly imitating the behaviours of the person they are observing and using that imitation as the basis of their motor-based predictions. It follows that they should be better at predicting recordings of their own behaviours than other people's behaviours. And in fact they are better at predicting recordings of themselves than others when throwing darts (Knoblich & Flach, 2001) or playing the piano (Keller, Knoblich, & Repp, 2007). To predict other people's behaviour, they must additionally make adjustments based on self-other differences (e.g., relating to arm trajectory or finger movements) and override the tendency to predict based on their own behaviour in the past.

Prediction-by-simulation may underlie prediction of speech (Pickering & Garrod, 2013; see also Dell & Chang, 2014). For it to occur, listeners covertly imitate what they hear and use it to determine the speaker's likely continuation based on what the listener would be likely to produce under the circumstances. There is considerable evidence that listeners use their own production systems to make predictions (Pickering & Gambi, 2018). For example, Drake and Corley (2015) presented participants with sentence fragments that predicted a particular completion (e.g., *tap* after *When we want water, we just turn on the …*). They found that when participants then named a picture phonologically related to the predicted word (e.g., *cap*), articulation diverged more from a control condition in which participants named the pictures without any sentence context, compared to when they named the predicted picture (e.g. *tap*) instead. Thus, predictions made during comprehension influenced later speech production, suggesting that prediction and production share a common mechanism. Additionally, Martin, Branzi, and Bar (2018) found that the N400 effect (associated with unpredictability) for articles in unexpected noun phrases (e.g. *a hat*) embedded in highly predictive contexts (e.g. *The king wore on his head …*) was reduced when participants simultaneously produced the syllable /*ta*/ than when they tapped their tongue or listened to their own voice producing /*ta*/. Thus, participants appear to be worse at predicting when they are simultaneously using their language production system.

The continuation that listeners would produce is in turn based on what they have produced in the past in similar situations. Importantly, as this prediction is based on their experience of speaking, it would retain traces of the way they speak (i.e., their own stylistic idiosyncrasies). In other words, we propose that listeners predict not only the upcoming word, but also the way that the word will be uttered – a way that is related to how they would themselves utter that word. Such predictions could therefore cover a variety of aspects of the speech spectrum, including those relating to speech style. While the listener's default model is based on their own speech, for predictions to be appropriate, they must be adjusted to account for self-other differences (Pickering & Gambi, 2018); the further the style of the speaker from that of the listener, the more adjustment is needed. This means that with minimal time to model a new speaker, listeners should be better at predicting speech that is stylistically similar to their own (and thus similar to their default model) compared to speech that is stylistically dissimilar to their own (and thus dissimilar from their default model).

Importantly, speech style is made up of a range of speech parameters relating to the spectrum and duration of speech (Bradlow, Torretta, & Pisoni, 1996; Gimson & Ramsaran, 1970). However several parameters are particularly promising in the study of simulation: specifically, those shown to converge between interlocutors over time. Such convergence indicates a link between perception and production, and has been demonstrated for features of speech including interlocutors' rates of talking (Finlayson, Lickley, & Corley, 2012; Giles, Coupland, & Coupland, 1991), fundamental frequencies (Gijssels, Casasanto, Jasmin, Hagoort, & Casasanto, 2016; Gregory & Webster, 1996), intensities (Gregory & Hoyt, 1982), and accents (Babel, 2012). These parameters may therefore be particularly salient for prediction via simulation.

Prediction-by-simulation may provide an explanation of how prediction can help explain short turn intervals in conversation. Listeners could make predictions about a speaker's timing in a similar way to making predictions about content, using prediction-by-simulation to predict the speaker's turn-end. But as well as predicting the speaker's turn end, successful turn-taking requires that interlocutors prepare their response to that turn and initiate it on time. Predicting the timing of a partner's turn may thus allow interlocutors to prepare and initiate their response to a partner's speech more quickly. However, comprehenders' processing load increases at the end of the speaker's turn (Boiteau, Malone, Peters, & Almor, 2014), presumably because of the difficulty of preparing their own utterance while still processing the speaker's. It is therefore also possible that having to concurrently prepare a response interferes with interlocutors' ability predict the speaker's turn-end or their ability to make appropriate use of this prediction.

### 1.3 Current study

In this study, we investigate the role of simulation in prediction of a speaker's turn-end and initiation of a response. Participants recorded a set of spoken utterances containing a statement and a question, in two sessions about three months apart (to allow analysis of potential memory effects, discussed below). They then returned about three months later again, and listened to their own recordings, those of a speaker independently rated as similar in style to themselves, and those of a speaker independently rated as dissimilar in style to themselves. In this session they took part in two tasks. In the button-press task, they pressed a button when they predicted that the speaker's turn would end. In the spoken-response task, they produced a spoken response to the question. We therefore investigated the effects of simulation on turn-end prediction itself, and also on the processes involved in producing a response.

According to prediction-by-simulation, comprehenders should be better at predicting the timing of utterances that are more similar to their own utterances than those that are less similar to their own utterances. We used raters to identify a speaker whose utterances were very similar to a comprehender and a speaker whose utterances were very dissimilar to a comprehender. According to prediction-by-simulation, the comprehender should be better at predicting the timing of the similar versus the dissimilar speaker. We also assumed that the comprehender should be better at predicting his or her own utterances than those of the dissimilar speaker. Finally, if the similar speaker's utterances deviate substantially from the comprehender's utterances, then the comprehender may also be better at predicting his or her own utterances than those of the similar speaker. Due to the need for dissimilar speakers, we recruited participants across a wide age-range and did not require native English speakers.

We additionally analysed change in performance across the two recording times to determine whether differences in responses to self-compared to the other recorded speakers were due to simulation, or simple memory of one's own speech. If they were due to memory, predictions of the comprehender's own speech would be better for stimuli recorded more recently (i.e., the second session) than less recently (i.e., the first session).

## 2 Materials and methods

### 2.1 Participants

Thirty-one participants took part in this study (17 female, 28 native English speakers). Participants were aged 19–67 years (*M* age = 39.9, SD = 17.0). Each participant was paid £52 for taking part. This study was approved by the University of Edinburgh Psychology Research Ethics Committee and informed consent was acquired from all participants.

### 2.2 Items

We constructed 120 two-sentence items such as (a) and (b) below (see Supplementary material for full list)

a. I like watching different sports on the TV. Do you like to watch football?
b. I love travelling. Have you ever visited the city of Paris?

To develop these items, we recruited 20 further participants from the same population as the main experiment (*M* age = 43.1 years, 12 female, 16 native English speakers). These participants were presented with 150 item candidates with the final word removed in an on-line task, and were asked to complete them with a single word. Cloze predictability was calculated for each response given by participants (i.e., the percentage of participants providing that particular completion). We selected items with the most frequent completion, and excluded items in which two completions were equally frequent, items in which the most frequent completions contained a word used in the context, or items with the same completion as another item.

Each item consisting of a statement followed by a question and was 7–37 words long. For 60 items, the final word of the question was highly predictable (30–80% Cloze, as in 1); for 60 items, it was moderately predictable (10–30% Cloze, as in 2). It was always more predictable than any alternative completion (i.e., it was the most common response in the pre-test). Two different sets of 60 items were created (sets A and B), each containing 50% highly predictable endings and 50% moderately predictable endings (see Supplementary material). This meant that items in each set had a range of predictabilities (and subsequently that prediction also varied within each condition).

### 2.3 Procedure

#### 2.3.1 Participant procedure

In sessions 1 and 2, participants recorded the two sets of items, 90 days (±14 days) apart (set order counterbalanced between participants). A further 90 days later (±17 days) in session 3, participants listened to and predicted the end of 60 utterances (button-press condition), and listened to and produced a spoken response to the other 60 utterances (spoken-response condition). In this final session, both the items in the button press vs spoken response conditions, and the items presented by each type of speaker, were counterbalanced.

To explain the counterbalancing, in each of the button press and spoken response conditions half of the items were taken from set A and half were taken from set B, counterbalanced between participants, so that each item was presented in the button press condition for half of the participants and in the spoken-response condition for the other half. (As participants recorded the two sets of items in different sessions, half of the items in each condition were from recording session 1 and half from recording session 2.) Furthermore, in each of the button press and spoken response conditions, an equal number of stimuli were presented in each recorded speaker's style (i.e., 20 in their own speech; *self* condition, 20 by a similar speaker; *similar* condition, and 20 by a dissimilar speaker; *dissimilar* condition). Items presented by each type of recorded speaker were counterbalanced between participants, so that each item was presented in the self condition for one third of participants, the similar condition for one third of participants, and the dissimilar condition for one third of participants. The order of the stimuli was individually randomized.

Items were presented on an 18 inch monitor positioned centrally behind the keyboard, approximately 60 cm from the participant. Participants listened to the items over a pair of ‘Beyerdynamic DT 109’ headphones with a microphone that was also used for recording the items. The experiment was run using E-Prime 2.0 and an SR-box with a microphone positioned 5 cm from the participant's mouth for recording the onset of vocal responses in the spoken response experiment.

#### 2.3.2 Rating procedure

The recordings from session 1 were used by two independent raters (one experimenter, one voluntary assistant) to choose one speaker similar to each participant and one speaker dissimilar to each participant. As the order of lists recorded each session was counterbalanced between participants (i.e., half recorded set A first, half recorded set B first), participants were compared only with the half of the participants that recorded the same list as them in session 1.

To rate participants' similarities, we arbitrarily selected five utterances (indicated by an asterisk in the supplementary material) and had the raters listen to each speaker's production of these utterances (i.e., 31 × 5 = 155 utterances). The raters' task was to choose the overall five most similar and the five most dissimilar speakers to each speaker, with volume, accent, and tone as suggested criteria. For example, for utterance 1 by speaker 1, the raters chose the most similar and the most dissimilar speakers (i.e. other participants) of utterance 1. They repeated this procedure for the other four utterances for speaker 1. They then moved on to speaker 2, and so on. Therefore, one rater could choose another speaker as similar or dissimilar to the current speaker a maximum of five times (based on all five utterances).

This procedure kept the task manageable to the raters, but meant that the similarity judgements were based on several utterances. The number of times each participant was chosen as most similar or most dissimilar was then summed across all five utterances for both raters, and the participant with the highest score was selected. In all but two cases, the speaker finally selected as the most similar, or most dissimilar, to the participant was included in both raters' lists of their top five. In the two cases where that wasn't the case, they were in the raters' top six. If a specific participant was selected as the most similar/dissimilar speaker to more than three participants, the second most similar/dissimilar speaker was selected for additional participants instead.

To elucidate the ways in which the selected similar and dissimilar speakers were similar or different to the participants, we analysed the five utterances used for rating in terms of average fundamental frequency (Hz), speech rate (syllables/second), and intensity (dB). By comparing how much each participant's speech properties differed from those of the speaker selected as most similar and those of the speaker selected as most dissimilar, we found that ratings were at least partly based on fundamental frequency information. In other words, the partners identified as most similar were closer in pitch to the participant than the partners identified as most dissimilar (similar partner difference in f0 = 21.5 Hz, dissimilar partner difference in f0 = 77.9 Hz, t(30) = −6.884, *p* < .001). However, we did not find similar partners to be closer to the participant than dissimilar partners in terms of speech rate or intensity (similar partner difference in speech-rate = 0.87 syllables/s, dissimilar partner difference in speech-rate = 1.07 syllables/s, t(30) = −1.352, *p* = .19; similar partner difference in intensity = 5.47 dB, dissimilar partner difference in intensity = 6.24 dB, t(30) = −0.859, *p* = .40). Note direction of difference was not considered (i.e., similarity was absolute distance from participant).

#### 2.3.3 Button press task

Participants were instructed to: ‘Press the button (using your dominant hand) when you believe the question will end. Do not wait until the speaker has finished the question and stopped speaking. Instead, you should press the button as soon as you expect the speaker to finish.’ This instruction was the same as used by Corps, Crossley, et al. (2018). On each trial, the participant was presented with a fixation cross until they pressed a button on the SR-box to begin listening to the utterance. When the participant pressed the button again to make their prediction, the speech stopped and the trial ended. (The speech did not continue after the button press, as we judged that participants might then wait for the end before responding). If the participant did not press the button by 5 s after the end of the stimulus, the trial was recorded as being missed. The procedure then repeated.

#### 2.3.4 Spoken-response task

Participants were instructed to ‘Answer as quickly as possible. Do not wait until the speaker has finished the question and has stopped speaking. Instead, you should answer as soon as you expect the speaker to finish the question.’ Again, this instruction was the same as Corps, Crossley, et al. (2018). They were specifically asked to ‘please respond with more than one-word answers, as if having a conversation’. When the participant spoke, the SR-box recorded onset time, the speech stopped, and the trial ended (as in the button press experiment). If the participant did not speak within 5 s of the end of the stimulus, the trial was recorded as being missed. The procedure then repeated.

### 2.4 Analysis

We analysed the data from the two tasks separately. Each task had a 3 (recorded speaker: self, similar, dissimilar) × 2 (recording time: 1 = 6 months prior, 2 = 3 months prior) design. Any trials in which the participant did not respond were removed (7.15% of trials). Response times were relative to the stimulus offset even if they responded prior to its actual occurrence (i.e., were negative for early responses and positive for late responses). Each participant's mean and standard deviation was calculated for each task and recorded speaker separately (i.e., separately for self, similar, and dissimilar speaker in each of the button press and spoken response tasks). Trials with responses >2.5SD from participants' means were then removed (1.85% of trials). In total, 9.84% of remaining responses occurred prior to stimulus offset.

Analyses were conducted in R using the package lme4 (Bates, Maechler, Bolker, & Walker, 2014) and lmerTest (Kuznetsova, Brockhoff, & Christensen, 2015). Linear mixed effect models included full fixed factor specification, with fixed effects comprising recorded speaker, recording time, and their interaction. Only a random effect of speaker was included in each model due to non-convergence of more complex models. Significant predictors from the model are reported using ANOVA with degrees of freedom determined using the Satterthwaite method. Subsequent pairwise comparisons were performed using the package emmeans (Lenth, 2018), with Tukey adjustment.

## 3 Results

**Fig. 1:**
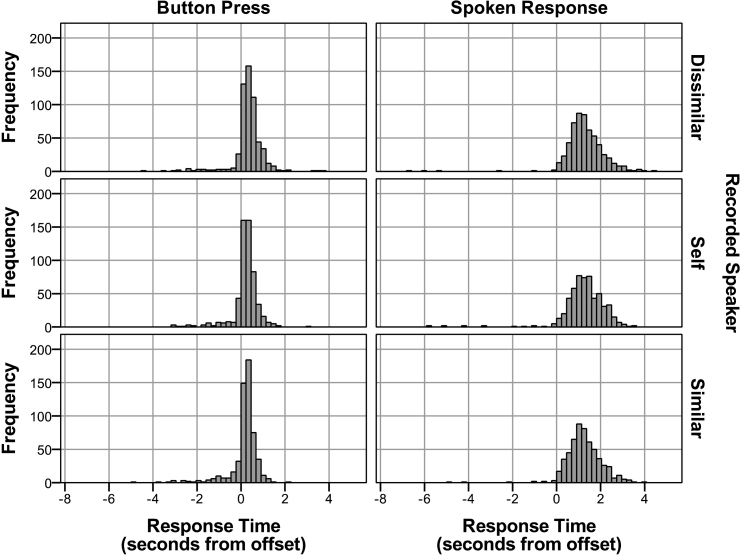
Response-time distributions by recorded speaker for each of the button press and spoken response conditions (following outlier removal).

For the button press condition, the mean response was 234 ms after stimulus offset, with a standard deviation of 655 ms. For the spoken-response condition, the mean response was 1272 ms after stimulus offset, with a standard deviation of 898 ms (see Fig. 1).

**Fig. 2:**
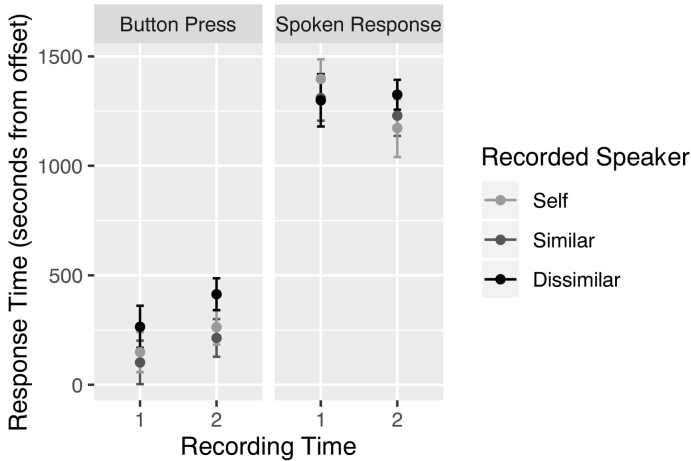
Mean response by recorded speaker and recording time (1 = six months prior, 2 = three months prior) for each of the button press and spoken response conditions.

Button-press task (Fig. 2, Table 1): There was a main effect of Recording Time (F(1,84) = 20.58, *p* < .001), with participants responding 125 ms more quickly for stimuli recorded six months prior than three months prior. Importantly, there was a main effect of Recorded Speaker (F(2,28) = 10.53, *p* < .001), indicating that participants' responses were affected by speaker identity. More specifically, participants responded later to the dissimilar speaker than their own speech (difference = 133 ms; *p* = .018), and later to the dissimilar speaker than the similar speaker (difference = 181 ms; *p* < .001). Responses did not differ between self and the similar speaker (difference = 48 ms; *p* = .57). No other effects were significant.

**Table 1:** Mean (and standard deviation) of reaction times (ms) in the button press and spoken response conditions for items recorded 6 months and 3 months prior to the third session.

	Button press		Response initiation	
	6 months prior	3 months prior	6 months prior	3 months prior
Self	149 (494)	263 (426)	1397 (490)	1173 (726)
Similar	102 (536)	214 (462)	1310 (567)	1229 (506)
Dissimilar	265 (516)	414 (399)	1300 (656)	1325 (375)

Response Initiation task (Fig. 2, Table 1): There was a marginal effect of Recording Time (F(1,87) = 3.42, *p* = .068), with participants showing a trend to respond more quickly for stimuli recorded three months prior than six months prior (difference = 93 ms). No other effects were significant.

We ran several control analyses to ensure findings were robust. First, we note that the experiment included three non-native participants. We included such participants, just as we included participants with a broad age range, to increase potential dissimilarity among participants. When removing the non-native participants all results were statistically equivalent. Second, we note that the above analysis focused on the relative timing of responses, which could allow negative and positive responses to cancel out. An analysis of the absolute difference between the participant's response and the stimulus offset was thus conducted (models reduced to minimal random effect structures due to lack of convergence). For the button press condition, the effect of recorded speaker was replicated, but the effect of recording time was not. For the spoken response condition, the effect of recording time moved from being marginal to being significant.

## 4 Discussion

We asked participants to respond to an utterance ending either by pressing a button or producing a spoken response. They pressed a button more quickly, and closer to the actual offset, if the utterance had been produced either by themselves or by a speaker rated similar to themselves than it had been produced by a speaker rated dissimilar to themselves. There were no comparable effects when they produced a spoken response.

We tested the theory that comprehenders simulate the speaker's utterance and then use that simulation to predict the speaker's timing. Specifically, that they covertly imitate the speaker's utterance and construct the representations that they would use if they were speaking at that point themselves. In this way, they can predict the time at which they would stop speaking (i.e., end their turn) if they were the speaker. But of course such prediction is less good if the comprehender speaks differently from the speaker currently talking, for example if their speech pitch is different. Thus comprehenders are less good at predicting the speech of speakers who are dissimilar from themselves than speakers who are similar to themselves (or indeed their own speech). Speakers attempt to adjust for self-other differences (see Pickering & Gambi, 2018) in order to determine when the speaker is likely to end, but adjustment is difficult. Less adjustment is necessary for speakers similar to themselves than speakers dissimilar to themselves.

We showed that one's idiosyncratic motor experience leads to increased prediction when listening to speech. Participants predicted dissimilar speakers (who differed from the participants in terms of fundamental frequency) more poorly than either themselves or similar speakers. We propose that the lack of a difference between the self and similar conditions reflects genuine similarity – every participant has another person who is very similar to themselves. As we used the other person in our sample who spoke most similarly to the participant (subject to the limitations of our rating method), we propose that the self and similar conditions did not differ sufficiently for us to demonstrate a difference between them. The fact that speakers do not perceive their speech exactly the same when listening to a recording as they do when speaking (e.g., due to bone conductance), may also contribute to the lack of difference between self and similar speakers. However, it is of course possible that our experiment was insufficiently sensitive.

We compared responses to stimuli recorded at different times to address whether these differences were due to memory effects. In the button-press study, participants responded more quickly (and therefore more accurately) for stimuli recorded six months prior compared with three months prior, with the opposite trend being seen in the spoken response experiment. We have no explanation for these findings, but note that they provide no evidence that our results are based on better memory for more recently recorded speech (though note that both the three and six month conditions would have relied on long-term memory representations, if the recordings were stored at all).

Our study revealed no difference among Recorded Speaker conditions in the spoken-response experiment. It is possible that we have simply failed to detect a real effect, but a more interesting possibility is that participants have more difficulty in this task because they have to plan and initiate a (free) response, and that such activities interfere with the ability to precisely determine turn-ends (or supersede the use of turn-end prediction during response production). Moreover, their focus in this task is on the content of the speaker's utterance rather than its timing (due to having to verbally respond), so they may pay less attention to the information that is most useful for determining turn-end.

While we used subjective judgements of similarity to identify similar and dissimilar speakers in this study, it is interesting that our (exploratory) analysis of the acoustic speech parameters showed that these ratings were at least partly based on differences in fundamental frequency. Given the limited information on which the raters made their similarity judgements (i.e., five utterances), an interesting possibility is that acoustic parameters of the voice that relate to physical features of the speaker such as size (Kreiman & Sidtis, 2011) are initially the most salient. It is, however, also possible that there was simply not enough variability in speech rate or intensity to differentiate speakers easily based on those parameters. Nonetheless, the fact that two independent raters showed such consistency, and that their ratings could be specifically tied to an objective measurement, is reassuring.

It is interesting to compare our findings with prior studies investigating prediction in language. In the study by De Ruiter et al. (2006), they reported earlier responses for unintelligible than intelligible utterances, whereas we saw earlier responses to self and similar speakers than dissimilar speakers. These findings may appear contradictory, but note that both we and de Ruiter et al. found responses to the more predictable stimuli (i.e., self/similar recordings, or intelligible utterances) to be closer to the stimulus offset. The timing of our participants' responses, however, were on average positive (i.e., late), whereas their participants' responses were on average negative (i.e., early).

Our study can also be compared to a similar study that focused on predictions in music. Keller et al. (2007) found that pianists were more synchronised when playing a musical duet with a recording of themselves than with a recording of another musician. Given that musical synchronisation inherently relies on monitoring and predicting one's duet partner in order to play at the same time as them, this suggests that simulation processes facilitate prediction of outputs produced in a similar style to one's own in both linguistic and musical domains.

In conclusion, we have investigated how listeners predict when speakers will complete their utterances. We show that listeners are better at making such judgements when listening to their own utterances or utterances produced by speakers similar to themselves than when listening to utterances produced by speakers dissimilar to themselves. We argue that this is because they simulate the utterances by covertly producing them and using that covert production to run the utterances on ahead, and that such simulation is affected by their similarity to the speaker.

## Funding sources

This work was supported by the 10.13039/501100000275Leverhulme Trust [grant number RPG-2017-239 awarded to MJP and LVH]; the 10.13039/501100000265Medical Research Council [grant number MR/S003576/1]; and the 10.13039/501100000589Chief Scientist Office of the Scottish Government. Funding sources had no role in experiment design or analysis.

## CRediT authorship contribution statement

**Lauren V. Hadley:** Conceptualization, Formal analysis, Writing - original draft. **Nina K. Fisher:** Methodology, Investigation, Data curation, Writing - review & editing. **Martin J. Pickering:** Supervision, Writing - review & editing, Funding acquisition.

## Declaration of competing interest

None.

## Appendix A Supplementary data

Supplementary material Image 1
