# Supplementary material for: Listeners are better at predicting speakers similar to themselves
Source: Acta Psychol (Amst). 2020 Jul;208:103094. doi: 10.1016/j.actpsy.2020.103094 (PMC7408002; doi:10.1016/j.actpsy.2020.103094)
Supplement: Supplementary file 1 — Supplementary material [file mmc1.docx]

**Supplementary material: Stimuli list with final question fragments and cloze probabilities**

*** Stimuli used in rating procedure**

**Stimuli List 1**

| **Stimuli No.** |  | Completed Question Fragment | Cloze Probability |
| --- | --- | --- | --- |
| **1** | I have been to the hospital a few times. Have you ever had to visit the hospital after injuring your | leg | 20 |
| **2** | I try not to snack too much during the day. Is your favourite snack | crisps | 30 |
| **3** | I enjoy reading different things. Do you read | books | 25 |
| **4** | I went to London last year. When you visited, did you see your | friend(s) | 30 |
| **5** | I really like Disney movies. Is your favourite animated character | Bambi | 20 |
| **6** | I heard you are going away on holiday soon. When you visit the USA, would you like to visit | Florida | 10 |
| **7** | I heard you are going to London next week. When you are there, are you going to visit the | museum(s) | 25 |
| **8** | We had a great time playing games at the party. Is your favourite game | charades | 20 |
| **9** | There is nothing to watch on TV. Do you have any suggestions for any good | films | 25 |
| **10*** | I don't know where to go on holiday next summer. Do you think I should go to | Italy | 15 |
| **11** | I enjoy drinking warm drinks when it is cold outside. Do you like | coffee | 30 |
| **12** | I like to use watercolours for drawing and painting. Do you prefer to use | oils | 20 |
| **13** | I think I am going to get a change at the hairdressers. Do you think I should get my hair | cut | 30 |
| **14** | I am thinking of applying to do a Masters degree. Do you think I should apply to | Cambridge | 20 |
| **15** | I have forgotten when the meeting is. Is it on | Monday | 25 |
| **16** | There are a lot of different courses to choose from. Do you think you made the right choice studying | psychology | 15 |
| **17** | I like lots of different bands. Is your favourite band | popular | 15 |
| **18** | I like to be active. Is your favourite sport | football | 25 |
| **19** | The news reported that it is going to be really cold tomorrow. Are you going to go to the | park | 20 |
| **20** | Some people get up really early. Do you wake up before | six | 30 |
| **21** | The driest month in Scotland is May. Is the wettest month | April | 30 |
| **22** | My favourtie type of pasta shape is macaroni. Is your favourite | spaghetti | 30 |
| **23** | I have two great fears. Are you afraid of | spiders | 30 |
| **24** | Sometimes I have cravings for really bizarre foods. Do you ever crave | chocolate | 15 |
| **25** | Someone asked me what appliance I could not live without but I was unsure. Could you live without your | TV | 15 |
| **26** | Someone asked me the other day what super power I would have if I could choose. Would you rather be invisible or | strong | 30 |
| **27** | Someone asked me what job I would like to try for a day, but I really couldn't make up my mind. I was thinking of being a pilot, but what do you think about being a | astronaut | 15 |
| **28** | I don't really like watching horror movies. Do you prefer watching horror or | comedy | 25 |
| **29** | I have been on holiday to lots of different warm places. Have you ever been to | Spain | 20 |
| **30** | I am not sure what the norm is. Do most people have two | children | 20 |
| **31*** | I try to get away on holiday at least once during the year. Would you like to go on holiday to a warm | country | 45 |
| **32** | I eat lots of fruit for breakfast. Is your favourite fruit a | banana | 50 |
| **33** | You always seem to be really busy at the weekends. Do you often see your | family | 40 |
| **34** | Your train will be leaving soon. When will you | arrive | 40 |
| **35** | I really like going swimming during the summer. Have you ever been swimming in the | sea | 60 |
| **36** | I have not seen you for a long time. Shall we meet up and go for | coffee | 40 |
| **37** | You have been really busy recently. When are you next | free | 60 |
| **38** | I like watching different sports on the TV. Do you like to watch | football | 35 |
| **39** | You said you were going to go out for lunch. Did you find something tasty at the | restaurant | 35 |
| **40*** | You said you need to buy a lot of things. Would you like to go to the | shops | 55 |
| **41** | The living room is really cold. Shall I get you a | blanket | 60 |
| **42** | I would love a drink. Will you please get me some | water | 55 |
| **43** | I am looking forward to my sister's party. Have you found something good to | wear | 65 |
| **44** | If we are going to the cinema, we should book tickets. Do you have a preference for where we | sit | 75 |
| **45** | We live by the coast so when the weather is good we can spend the whole day at the beach. Do you enjoy the | seaside | 35 |
| **46** | The plane will be delayed by three hours. Do you have any suggestions for where we can | eat | 45 |
| **47** | There are not many cities to visit in Scotland. Have you ever been to | Edinburgh | 40 |
| **48** | I am going to go to the beach. Do you think I should bring a | towel | 65 |
| **49** | I don't think I have a favourite colour. Is your favourite colour | blue | 55 |
| **50*** | My extended family is quite small. Do you have many | siblings | 35 |
| **51** | It is a really cold night. I am not sure if I will go for a | walk | 60 |
| **52** | I am really hungry. Shall we go to a | restaurant | 35 |
| **53** | I have a few friends that speak another language. Do you also speak | French | 40 |
| **54** | I am not sure what my favourite one would be. Is your favourite flower a | rose | 35 |
| **55** | I never know what time it is. Can you check the time on your | watch | 55 |
| **56** | I have just put the kettle on to make some tea. Would you like a | cup | 65 |
| **57** | It would take me about 40 minutes to walk home. Do you think I should go by | bus | 60 |
| **58** | I find the front crawl the easiest way to swim. Do you prefer | breaststroke | 35 |
| **59** | There is a Monday night five-a side-team. Do you play | football | 75 |
| **60*** | I enjoy going to see live music shows. Do you prefer going to see live music or live | theatre | 50 |

**Stimuli List 2**

| **Stimuli No.** |  | Completed Question Fragment | Cloze Probability |
| --- | --- | --- | --- |
| **1** | I have broken a number of bones in my body. Have you ever broken your | wrist | 20 |
| **2** | I love travelling. Have you ever visited the city of | Paris | 15 |
| **3** | I have a number of classes. Do you have any lectures on | Monday | 15 |
| **4** | I like all different types of games. Have you ever watched a game of | football | 25 |
| **5** | I love the outdoors. Would you like to go for a walk in the | woods | 30 |
| **6** | I enjoy playing games with friends and family. Have you ever played a game of | monopoly | 15 |
| **7** | I like to learn about different animals. Have you ever seen a wild | boar | 25 |
| **8** | Once I find what I like, I normally stick with it. In the past, have you had lots of different | hobbies | 25 |
| **9** | I have a few photos. Would you like to see a picture of my | daughter | 20 |
| **10*** | I get on with most people. Are there a lot of people in your | family | 25 |
| **11** | I think it is going to rain all day tomorrow. Do you think I should pack a | umbrella | 25 |
| **12** | I think it will be really good. Are you looking forward to | it | 25 |
| **13** | I manage to hurt myself all the time. Have you ever hurt your | leg | 15 |
| **14** | I do not have many talents. In your opinion, do you think you are a good | singer | 20 |
| **15** | I eat far too much sugar. Is chocolate your favourite | treat | 30 |
| **16** | I am going to buy some new clothes. Should I buy a nice dress for my | birthday | 25 |
| **17** | It looks like you know a lot of people. Are you in a | club | 30 |
| **18** | I do not really have a favourite. Is your favourite food | spicy | 25 |
| **19** | I like lots of bright colours. If you could, would you change the colour of the | bedroom | 15 |
| **20** | I like to do a variety of hobbies. Have you ever tried | knitting | 20 |
| **21** | You like trying out different places right? Where should I go for | dinner | 25 |
| **22** | I would love to build my own house one day. If you could design your own house, would it be | big | 25 |
| **23** | My friend is quite anxious about a lot of things. Do you get nervous about | flying | 30 |
| **24** | People study some really obscure subjects. Would you consider studying | anthropology | 10 |
| **25** | I am not sure when it was that I decided to work in this job. Did you always know that you wanted to be a | doctor | 20 |
| **26** | I am thinking of re-painting my kitchen. Do you think a good colour would be | blue | 20 |
| **27** | To make your food interesting, you can use a variety of spices. Do you like | chilli | 25 |
| **28** | I really enjoy visiting foreign countries. Have you ever been to | japan | 15 |
| **29** | I am thinking of buying a car. Do you think an Audi is better than a | BMW | 25 |
| **30** | I am going travelling in the summer. Do you think I should take a | coat | 15 |
| **31*** | I love discovering new music. When you buy music to listen to, do you buy CD's or | download(s) | 50 |
| **32** | I am a bit careless with my electronic devices. Have you ever broken your | phone | 65 |
| **33** | Most of the sports we play today have been around for a really long time. Do you think the oldest sport still played is | football | 40 |
| **34** | I love travelling, but I am not sure what the best way to travel is to get around Europe. Do you think the best way to travel is by | train | 65 |
| **35** | I really love living here. If you could only choose one city to live in for the rest of your life, would you choose | London | 45 |
| **36** | I don't really have a favourite flavour. Is your favourite ice-cream flavour | vanilla | 50 |
| **37** | My friend asked me what I would take with me if I could only take one thing to a deserted island but I couldn't make up my mind. Would you take a | book | 35 |
| **38** | When I first meet people, I always notice the way that they shake hands. Is the first thing you notice about people the way they | smile | 40 |
| **39** | When I go on holiday, I like to do different things. Do you prefer holidays on the beach or in the | city | 35 |
| **40*** | I like listening to different genres of music. Do you like | jazz | 35 |
| **41** | I really like bright colours. Is a banana the same colour as a | lemon | 65 |
| **42** | I need to run to the shops. Do you need to go to the supermarket to buy some | milk | 35 |
| **43** | I like going out in the evenings. Do you enjoy going to the | cinema | 50 |
| **44** | I like the spring. During spring, do you like spending time at the | park | 65 |
| **45** | I would really like to get a pet. If you could get a pet, would you like to get a | dog | 50 |
| **46** | I am thinking about what I need to buy today. Should I buy a new suit for the | wedding | 55 |
| **47** | I am not scared of very many things. Are you scared of | spiders | 50 |
| **48** | My friends just had a baby. Do babies often cry when they are | hungry | 70 |
| **49** | My house is always busy. Do you live in a house with other | people | 80 |
| **50*** | I love reading. Do you read | much | 35 |
| **51** | I am going to my friend's party tonight. Do you think I should bring vodka or | gin | 35 |
| **52** | I took my car into the garage yesterday. Do you think it needs to get new | tyres | 45 |
| **53** | I always get burnt when it is this sunny. Could I borrow a | hat | 60 |
| **54** | I have just finished reading a Shakespeare play. Have you ever read | hamlet | 50 |
| **55** | My friend's dinner party is tomorrow and he asked me to bring some cheese for the cheese platter. Do you think I should buy | brie | 45 |
| **56** | Haggis is considered the national dish of Scotland. What is the national dish of | England | 35 |
| **57** | I was recently asked what my fantasy dream house would be. If you could, would you make a house out of | wood | 45 |
| **58** | I cannot speak any other languages. Would you like to learn | French | 35 |
| **59** | My auntie plays the lottery every single week. If you suddenly won the lottery, would you buy a | house | 45 |
| **60*** | I don't know what I should make for dinner. Should I make some | pasta | 40 |
